# Effects of a 10-Week Combined Coordination and Agility Training Program on Young Male Soccer Players

**DOI:** 10.3390/ijerph181910125

**Published:** 2021-09-26

**Authors:** Francisco Tomás González-Fernández, Hugo Sarmento, Alfonso Castillo-Rodríguez, Rui Silva, Filipe Manuel Clemente

**Affiliations:** 1 Department of Physical Activity and Sport Sciences, Pontifical University of Comillas, 07013 Palma, Spain; francis.gonzalez.fernandez@gmail.com; 2 SER Research Group, Pontifical University of Comillas, 07013 Palma, Spain; 3 Faculty of Sport Sciences and Physical Education, University of Coimbra, 3000-248 Coimbra, Portugal; hg.sarmento@gmail.com; 4 Research Unit for Sport and Physical Activity (CIDAF), 3000-248 Coimbra, Portugal; 5 Department of Physical Education and Sport, University of Granada, 18010 Granada, Spain; acastillo@ugr.es; 6 Escola Superior Desporto e Lazer, Instituto Politécnico de Viana do Castelo, Rua Escola Industrial e Comercial de Nun’Álvares, 4900-347 Viana do Castelo, Portugal; rui.s@ipvc.pt; 7 Instituto de Telecomunicações, Delegação da Covilhã, 1049-001 Lisboa, Portugal

**Keywords:** football, coordination, agility, training program, performance, athletes

## Abstract

The current literature has shown how working on coordination and agility produces effects on specific aspects in team sports. The purpose of this study was to examine the effects of a ten-week coordination training program applied to soccer on different tests that evaluate speed (30 m speed test), agility (Illinois Agility Test (IAT)) and lower body strength (countermovement jump (CMJ)). Forty U16 male soccer players from two nonprofessional teams (twenty in the control group (CG) (aged = 14.70 ± 0.47, body weight = 60.15 ± 8.07 kg, height = 1.71 ± 0.06 m) and twenty in the experimental group (EG) (aged = 14.50 ± 0.51, body weight = 58.08 ± 9.78 kg, height = 1.69 ± 0.06 m)) performed a combined coordination and agility program during 10 min every training day (3 days a week) for 10 weeks. The results of this study showed that coordination training produced adaptations in the power (CMJ of EG (*p* = 0.001)) and agility capacities (IAT of EG (*p* = 0.002)) of young soccer players, but not on speed performance at longer distances (CG, *p* = 0.20 and EG, *p* = 0.09). Despite the benefits of the training program, a combination of training methods that includes power, agility, speed, and strength can enhance such improvements.

## 1. Introduction

Preadolescence is a developmental stage characterized by major physiological changes in the musculoskeletal and neuromuscular systems [1,2]. Therefore, physiological adaptations in preadolescents after physical training are expected to be different compared to those in adults [3]. Considering that soccer training demands different fitness variables in players, it is expected to observe the improvement of different qualities after training interventions [1]. These improvements emerge from the high levels of physical capacity required to produce and maintain power during successive high-intensity efforts [4]. Considering that players must hold well-developed aerobic capacity while keeping optimized neuromuscular capacity, the task for coaches is not easy, since they need to work in multiple directions [5,6].

The interaction between physical qualities ensures the maximization of performance. As an example, high levels of lower-limb power are critical for ensuring better sprints [7,8,9,10]. Power is also dependent on strength, and for that reason is crucial strength training on soccer players [11]. However, physical capacity is not the only important thing to think about while developing players. In fact, coordination capacity also helps to improve multidirectional changes of direction (COD), strength and power application in specific soccer situations.

Agility is a central skill for performance and is defined as the execution of rapid movements [12]. In soccer, coordination training has generated adaptation in agility, which translates into the sum of different factors such as acceleration, deceleration, and COD [13]. Performance for these abilities is considered to be mainly influenced by physical components [14]. The external structure of a given movement (acceleration–brake–turn–acceleration capacity) is related to the nature of the muscle contraction required to produce it; therefore, lateral dominance must be determined in the early stages of maturation, as this influences the preferred side and the speed needed to make turns in COD [11]. This highlights the need for studies that consider the effect of coordination programs with soccer-specific elements on young soccer players.

During COD, strong, sudden braking is carried out, similar to that in CMJ executions. The height of the jump measured with the CMJ test depends on the take-off speed [15], which is dependent on the athlete’s ability to brake and suddenly accelerate [16]. The relationship between lower body strength assessed through the CMJ and both linear speed and COD in soccer has been investigated in depth [14,17,18,19]. Linear sprint, CMJ, and agility are independent locomotor skills [18]. In fact, agility is strongly related to motor coordination and depends on the neurophysiological organization of movement as well as the coordination of various degrees of freedom [20,21]. It is for this reason that a variety of exercises in coordination programs can benefit agility.

The main purpose of this research was to examine the effect of a 10-week coordination training intervention program with soccer-specific elements on developing-age soccer players. Other studies have shown that coordination is directly related to strength, agility and speed [22,23]. Thus, this type of intervention may be related to potential improvements in agility performance, compared to a control group without such an intervention [24,25]. The main hypotheses of the current study were that a combined coordination and agility training program would enhance (i) agility performance, (ii) power, and (iii) linear sprint.

## 2. Materials and Methods

### 2.1. Study Design

The study was conducted between February and April of 2021. At the time of these observations, the soccer players had completed between two and three months of training. A parallel two-group, longitudinal (pre, post) design was used with physical tests performed before (preintervention) and after (postintervention) the ten-week intervention period. The soccer players were assigned and matched into two groups, an experimental group (EG) and a control group (CG), based on the team of which they were a part. In order to investigate the effect of a 10-week coordination intervention program on young soccer players, those from the CG were asked to maintain their training routines, while those from the EG modified their training sessions by introducing ten minutes of coordination work in the warm-up.

### 2.2. Participants

A total of forty U16 young, male soccer players from two nonprofessional teams (twenty in the CG (aged = 14.70 ± 0.47, body weight = 60.15 ± 8.07 kg, height =1.71 ± 0.06) and twenty in the EG (aged = 14.50 ± 0.51, body weight = 58.08 ± 9.78 kg, height = 1.69 ± 0.06)) from the region of Baleares, Spain, were recruited from the city of Calviá, which has a population ranging from 30,000 to 50,000 inhabitants according to the National Institute of Statistics from the Spanish Government (http://www.ine.es/; accessed on 1 May 2021). These players trained twice a week (90 min per session) and played one match a week. The training sessions were based on technical and tactical content development (70% of training time), technical skill improvements (10% of training time), and general improvements in physical condition (20% of training time). Generally, training sessions comprised a warm-up, main part, and cooldown.

The participants’ parents obtained information about the main aims of the investigation and signed informed consent forms. All the soccer players in this study were treated according to American Psychological Association (APA) guidelines, which ensured the anonymity of participants’ responses. The study was conducted in accordance with the ethical principles of the 1964 Helsinki declaration for human research and was approved by the Research Ethics Committee of the Pontifical University of Comillas (2021/74). Inclusion criteria for the participants in this study were (i) reporting normal vision and no history of any neuropsychological impairments that could affect the results of the experiment, (ii) being an active player with federation license, (iii) not presenting any injuries during the previous two months, (iv) giving consent, and (v) participating in 85% of the training during the study period.

### 2.3. Procedure

#### 2.3.1. Preintervention

First, the team staff was informed about the objectives of the study, and the research team ensured that parents or guardians signed their informed consents after having received details of the possible benefits and risks of the study. Subsequently, the research team studied and planned every structure of training together with the coaches and physical trainers and both teams.

Secondly, the soccer players performed the tests following the same order, and with a minimum of 3 min of rest between tests. The anthropometry and countermovement jump were performed in a dressing room with a stable temperature of 21 °C and relative humidity of 51%. The Illinois Agility Test (IAT), the 30 m sprint test, and the Yo-Yo intermittent recovery test were also performed. The Yo-Yo intermittent recovery test—Level 1 (YYIRT1), only performed in the pretest, was performed on synthetic turf with a mean temperature of 18.2 ± 2.4 °C and relative humidity of 68 ± 5% in both moments of assessment. The maximal oxygen uptake (VO2max in mL/min/kg) was estimated by the next equation: VO2max = final distance (m) × 0.0084 + 36.4. (See Table 1, for more information). In addition, no rainy conditions occurred in the two moments of assessment, and the tests were performed at the same time of day (6:30 p.m.–8:30 p.m.). Subsequently, the CG staff were instructed to maintain their training routines and the EG was instructed about the coordination intervention program in their warm-up routines. Every training session and test was monitored by one main researcher and was applicated by the physical training coaches responsible for the teams, who were specially trained for accurate and reliable data recording (See Table 1, for more information).

#### 2.3.2. Intervention

The young soccer players completed a ten-week coordination intervention program, which was designed individually for the EG and supervised by the main researcher and physical coach responsible for this group, considering the capabilities that they worked on for each weekly match. We followed the guidelines of the American College of Sports Medicine (ACSM, 2010) to ensure the safety of the participants. A total of twenty proposed coordination and agility exercises, which lasted ten minutes in total, were integrated into the warm-up of the EG.

The proposal integrated the warm-up with a 10 min coordination circuit, which comprised the following tasks: (i) agility ladder (ten different series per session); (ii) multi-jump, unilateral jump, and bilateral jump (70–100 jumps per session); and (iii) zig zag between pikes, driving the ball, passing, and shooting at goal. The sessions increased in difficulty throughout the 10 weeks (see Table 1 for more information).

#### 2.3.3. Postintervention

After ten weeks, the CG and EG were evaluated at the same time of day as in the preintervention session (6:30 p.m.–8:30 p.m.), in a similar space and at a similar time with the same humidity conditions. Everything was the same as in the preintervention session except that the Yo-Yo intermittent recovery test was not performed. The soccer players in the CG were also given an opportunity to perform the same program as the experimental group after the study ended.

### 2.4. Measures

#### 2.4.1. Anthropometry

Height and body weight were collected at the two moments of assessment, at the same hour and at the same day of the week. Height was measured using a stadiometer (SECA 213, Birmingham, UK) to the nearest 0.1 cm, and players were asked to remove their shoes and other accessories that could influence the assessment. Players also had to be in a vertical and immobile position, with arms extended along the body, and look straight ahead in an upright position. For each measure, only one measurement was collected.

#### 2.4.2. Countermovement Jump

The CMJ was evaluated using the Chronojump-Boscosystem^®^ (Barcelona, Spain) developed by De Blas et al. [26], which revealed intraclass correlation test levels between 0.821 and 0.949 for measuring the height jump. This system was connected to a MacBook Pro (macOS Sur 11.1). The values were analyzed with a chronopic and recorded by Chronojump version 2.0.2. After a warm-up, participants performed the CMJ test three times on a contact platform with every load jump, with 20 s of recovery between attempts to minimize the effect of fatigue and three minutes between the different load jumps. The best jump (in cm) was considered as the final outcome. They were instructed to jump as high as possible after reaching a knee angle of ~90°. Participants were also instructed to keep their hands on the hips during the CMJ and to land with their legs extended with maximal feet plantar flexion. If any of these requirements were not met, the trial was repeated.

#### 2.4.3. Linear Sprint

The 30 m sprints were evaluated using the MySprint app [27]. In order to ensure a successful performance, the research team followed the protocol of Samozino et al. [27]. To ensure interobserver reliability, sprint time was analyzed by two independent observers and reflected almost perfect agreement, with no significant differences between raters observed [28]. The aim of this test was to run 30 m as fast as possible. The young soccer players were instructed to sprint at maximum speed and were given two attempts for each condition, with one minute of recovery between attempts to minimize the effect of fatigue. The research team recorded the best of the two attempts (measured in seconds by the MySprint app and Ipad Pro model A1673 (iOS 13.3.)) as the final outcome. A camera (HD of 1080p 60cps) was used to record and analyze all attempts.

#### 2.4.4. Illinois Agility Test

The length of the field was 10 m, while the width (distance between the start and finish points) was 5 m. Four cones were placed in the center of the testing area at a distance of 3.3 m from one another. Four cones were used to mark the start, finish, and two turning points. The subjects started the test lying face down, with their hands at shoulder level. The trial started on the “go” command, and the subjects began to run as fast as possible. The time was recorded using photocell timing gates (Microgate wireless Training Timer, Bolzano, Italy) with resolution of 1 thousandth of a second. Typical error of the photocells was between 0.04 and 0.06 s, while the smallest worthwhile change was between 0.11 and 0.17 s [29]. The trial was completed when the players crossed the finish line without having knocked any cones over. Two attempts were performed by every young soccer player, timed so that the last participant undergoing the test had the same amount of rest as the first person. The best score was considered as the final outcome and used for analysis [30,31,32].

#### 2.4.5. Statistical Procedures

For the treatment of the data, we used adequate statistical methods to calculate percentages and central and dispersion parameters (arithmetic mean and standard deviation). Descriptive statistics are represented as mean ± standard deviation (SD) with standard mean difference data. Tests of normal distribution and homogeneity (Kolmogorov–Smirnov and Levene’s, respectively) were conducted on all data before analysis. Paired sample *t*-test was used for determining differences as a repeated measures analysis (pre– post). Cohen d was the effect size indicator. To interpret the magnitude of the effect size, we adopted the following criteria: d = 0.20, small; d = 0.50, medium; and d = 0.80, large. To discover between-group differences, an ANCOVA test was performed using the pretest as a covariate and the times pre and post as factors. To interpret the magnitude of the effect size of ANCOVA we adopted the following criteria: *η_p_^2^* = 0.02, small; *η_p_^2^* = 0.06, medium; and *η_p_^2^* = 0.14, large. Data were analyzed using Statistica software (version 10.0; Statsoft, Inc., Tulsa, OK, USA).

## 3. Results

Descriptive statistics were calculated for each variable (Table 2).

First, we performed a paired measures *t*-test with participants’ body composition parameters (weight and BMI), which revealed no significant differences. We performed another, similar paired measures *t*-test with participants’ performance variables (CMJ, IAT, and 30 m) that also showed no significant differences (*p* = 0.18, *d* = 0.29; *p* = 0.22, *d* = 0.35; and *p* = 0.11, *d* = 0.17, respectively). Thus, data on composition parameters and muscular performance variables between CG and EG before the intervention of 10 weeks (at pretest) were similar and unremarkable.

**Figure 1:**
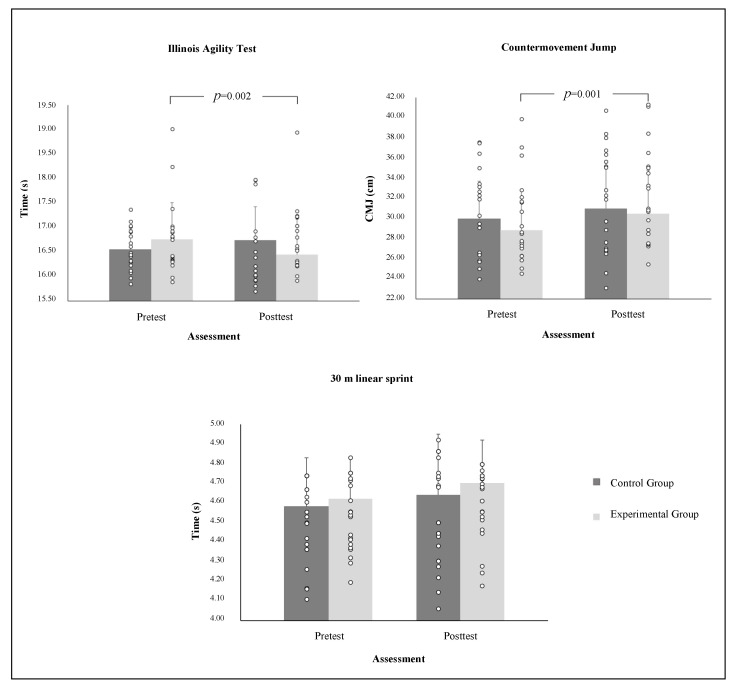
Performance variables before (preintervention) and after (postintervention) study period: a within- and between-group comparison. CG, control group; EG, experimental group.

A paired measures *t*-test with CG participants’ performance variables (CMJ, IAT and 30 m) revealed no significant differences (*p* = 0.09, *d* = 0.25; *p* = 0.12, *d* = 0.34; and *p* = 0.20, *d* =0.21, respectively). Data showed that participants did not improve significantly after 10 weeks. Lastly, a new repeated measures ANOVA with EG participants’ mean performance variables (CMJ, IAT, and 30 m) revealed a significant pre–post effect in CMJ and IAT (*p* = 0.001, *d* = 0.50 and *p* = 0.002, *d* = 0.41, respectively). However, data on the 30 m sprint did not reveal significant differences (*p* = 0.21, *d* = 0.37). (Figure 1). An ANCOVA test confirmed the effects between groups.

## 4. Discussion

The present study aimed to examine the effects of a ten-week coordination training intervention program on the physical performance of U16 soccer players. The results demonstrated that coordination training produced adaptations in the power and agility capacities of young soccer players, but not on speed performance at longer distances.

Considering CMJ test performance, the significant improvements in the EG after the coordination training intervention are in line with another study that showed similar significant improvements (43.17 vs. 44.01 cm; *p* < 0.05) in young athletes [33]. In fact, the authors of the aforementioned study also conducted a randomized controlled trial where the EG followed a 10-week coordination training intervention [33]. Another study also found similar improvements in CMJ performance after a speed, agility, and quickness (SAQ) training intervention [33]. However, the training interventions in these two studies [33,34] used different periodization approaches and different tasks constraints than the present study. Also, one of the authors used calisthenic exercises, including squats, during the warm-up, which may have interfered in the differences reported in power capacity [33]. Despite these methodological differences, all three studies showed that the implementation of coordination training produced improvements in jump performance, at least in younger populations.

Regarding agility test performance, the present study revealed significant reductions in the time to complete the IAT for the EG. In contrast to our findings, a study conducted on eighteen youth soccer players that analyzed the effects of a 6-week coordination training intervention on physical fitness revealed no significant differences between the pre- and posttests in agility performance [35]. However, the training intervention on the EG in that study comprised only ladder drills. The authors attributed the lack of differences in agility performance to the fact that the ladder drills used may have generated insufficient peak rate of force development to produce significant adaptations [35].

The use of coordination training interventions comprising dynamic and multidirectional movements has been related to improvements in agility and COD capacity in young and adult athletes, as reported in the present study [13,36]. This multidirectional coordination training may play an important role in improving agility, since some of the main skills involved in coordination and agility are the same. In fact, in concordance with our results, a study conducted on 20 elite U18 soccer players showed improved agility performance after a 6-week multidirectional-based training intervention [24]. Interestingly, another study that used a coordination training intervention with competition (e.g., using pairs) and without competition (e.g., individually), but using the same drills, found that coordination training with competition produced greater adaptations in agility after eight weeks [37]. Coaches should implement such training interventions to improve quickness and COD abilities if using a multidirectional approach that explores all vectors of movement.

Regarding the 30 m linear sprint test, neither the EG nor the CG presented significant differences from pre- to postassessments. This finding is in contrast with a study conducted on 100 junior soccer players that revealed significant improvements in the 30 m sprint test (2.15 vs. 2.07 s; *p* < 0.05) after an 8-week SAQ training intervention [34]. However, the latter study showed that players had greater improvements in the 5 m and 10 m splits, reinforcing the effectiveness of such a training intervention on speed and acceleration performance. The facts that the present study considered only the 30 m sprint and that our sample comprised nonelite athletes may explain the lack of differences found. Also, as soccer players typically perform shorter sprint distances (~5 to 15 m), it is possible that coordination training interventions produce improvements only in those distances related to acceleration [38]. Assessing 10 to 20 m sprint tests may be of greater use to track changes in performance after a coordination training intervention.

Coordination training intervention seems to improve young soccer players’ agility, power, and in some cases, speed performance [37]. However, the use of such an intervention is not enough to cover all of players’ physical needs. A recent study conducted on U17 soccer players compared the effects of a combined plyometric and short sprint with COD on physical variables [39]. Results revealed that the EG presented significantly greater improvements in jump, sprint, COD, repeated sprint ability, and static balance than the CG [39]. For these reasons, and given the characteristics of soccer, implementing mixed approaches in the training process, as well as introducing strength and power training, for U16 players is of paramount importance to build more resilient athletes [40,41,42].

## 5. Limitations and Future Lines of Research

The present study demonstrated that a multidirectional-based coordination training intervention can be beneficial in improving youth soccer athlete’s power and agility performance over a relatively short period of time. However, the present study also showed some limitations. One of the main limitations was related to the fact that we did not consider the 5, 10, or 20 m splits of the 30 m linear sprint test. No issues with adherence were found. One of the strengths of our findings is that coordination training showed auspicious benefits for power and agility in youth soccer players. Future studies should consider analyzing the first splits of a linear sprint test, as shorter distances (5 to 20 m) are related to acceleration, and longer distances seem to be independent of agility [20]. Another limitation was not including strength measures, as such measures may have helped to understand whether using a training intervention that included both accelerations and decelerations would be sufficient to produce strength adaptations in young soccer players.

## 6. Conclusions

This study aimed to examine the effects of a ten-week coordination training intervention program on the physical performance of U16 soccer players. The results of this study showed that coordination training produced adaptations in the power and agility capacities of young soccer players, but not in speed performance at longer distances. Although coaches can use coordination training to improve their players’ physical fitness, combining such training with other training methods such as strength and plyometrics would potentially increase chronic adaptations in agility, power, speed, and strength.

## Figures and Tables

**Table 1:** Intervention program. Description of 10-Week Combined Coordination and Agility Training Program on Young Male Soccer Players.

	W1			W2			W3			W4			W5			W6			W7			W8			W9			W10		
	P1			P3			P5			P7			P9			P11			P13			P15			P17			P19		
	TE	S	R	TE	S	R	TE	S	R	TE	S	R	TE	S	R	TE	S	R	TE	S	R	TE	S	R	TE	S	R	TE	S	R
**S1**	I			I			I			I			I			I			I			I			I			I		
	a b c d	6–8 6–8 6–8 6–8	36–48 12–16 12–16 6–8	a b c d	6–8 6–8 6–8 6–8	36–48 12–16 12–16 6–8	a b c d	6–8 6–8 6–8 6–8	36–48 12–16 12–16 6–8	a b c d	6–8 6–8 6–8 6–8	36–48 12–16 12–16 6–8	a b c d	6–8 6–8 6–8 6–8	36–48 12–16 12–16 6–8	a b c d	6–8 6–8 6–8 6–8	36–48 12–16 12–16 6–8	a b c d	6–8 6–8 6–8 6–8	36–48 12–16 12–16 6–8	a b c d	6–8 6–8 6–8 6–8	36–48 12–16 12–16 6–8	a b c d	10≈ 10≈ 10≈ 10≈	48–60 16–20 16–20 8–10	a b c d	10≈ 10≈ 10≈ 10≈	48–60 16–20 16–20 8–10
	II			II			II			II			II			II			II			II			II			II		
	a b	6–8 6–8	42–56 42–56	a b	6–8 6–8	42–56 42–56	a b c d	6–8 6–8 6–8 6–8	21–28 21–28 21–28 21–28	a b c d	6–8 6–8 6–8 6–8	21–28 21–28 21–28 21–28	a b c d e f	6–8 6–8 6–8 6–8 6–8 6–8	14–18 14–18 14–18 14–18 14–18 14–18	a b c d e f	6–8 6–8 6–8 6–8 6–8 6–8	14–18 14–18 14–18 14–18 14–18 14–18	a b c d e f g h	6–8 6–8 6–8 6–8 6–8 6–8 6–8 6–8	7–14 7–14 7–14 7–14 7–14 7–14 7–14 7–14	a b c d e f g h	6–8 6–8 6–8 6–8 6–8 6–8 6–8 6–8	7–14 7–14 7–14 7–14 7–14 7–14 7–14 7–14	a b c d e f g h i j	10≈ 10≈ 10≈ 10≈ 10≈ 10≈ 10≈ 10≈ 10≈ 10≈	7–14 7–14 7–14 7–14 7–14 7–14 7–14 7–14 7–14 7–14	a b c d e f g h i j	10≈ 10≈ 10≈ 10≈ 10≈ 10≈ 10≈ 10≈ 10≈ 10≈	7–14 7–14 7–14 7–14 7–14 7–14 7–14 7–14 7–14 7–14
	III			III			III			III			III			III			III			III			III			III		
	a c	6–8 6–8	36–48 36–48	a c	6–8 6–8	36–48 36–48	a c	6–8 6–8	36–48 36–48	a c	6–8 6–8	36–48 36–48	a b c d	6–8 6–8 6–8 6–8	16–24 16–24 16–24 16–24	a b c d	8≈ 8≈ 8≈ 8≈	16–24 16–24 16–24 16–24	a b c d	6–8 6–8 6–8 6–8	16–24 16–24 16–24 16–24	a b c d	6–8 6–8 6–8 6–8	16–24 16–24 16–24 16–24	a b c d	10≈ 10≈ 10≈ 10≈	16–24 16–24 16–24 16–24	a b c d	10≈ 10≈ 10≈ 10≈	16–24 16–24 16–24 16–24
	**P2**			**P4**			**P6**			**P8**			**P10**			**P12**			**P14**			**P16**			**P18**			**P20**		
**S2**	I			I			I			I			I			I			I			I			I			I		
	a b c d	6–8 6–8 6–8 6–8	36–48 12–16 12–16 6–8	a b c d	6–8 6–8 6–8 6–8	36–48 12–16 12–16 6–8	a b c d	6–8 6–8 6–8 6–8	36–48 12–16 12–16 6–8	a b c d	6–8 6–8 6–8 6–8	36–48 12–16 12–16 6–8	a b c d	6–8 6–8 6–8 6–8	36–48 12–16 12–16 6–8	a b c d	6–8 6–8 6–8 6–8	36–48 12–16 12–16 6–8	a b c d	6–8 6–8 6–8 6–8	12–16 12–16 12–16 12–16	a b c d	6–8 6–8 6–8 6–8	12–16 12–16 12–16 12–16	a b c d	10≈ 10≈ 10≈ 10≈	48–60 16–20 16–20 8–10	a b c d	10≈ 10≈ 10≈ 10≈	48–60 16–20 16–20 8–10
	II			II			II			II			II			II			II			II			II			II		
	a b	6–8 6–8	42–56 42–56	a b	6–8 6–8	42–56 42–56	a b c d	6–8 6–8 6–8 6–8	21–28 21–28 21–28 21–28	a b c d	6–8 6–8 6–8 6–8	21–28 21–28 21–28 21–28	a b c d e f	6–8 6–8 6–8 6–8 6–8 6–8	14–18 14–18 14–18 14–18 14–18 14–18	a b c d e f	6–8 6–8 6–8 6–8 6–8 6–8	14–18 14–18 14–18 14–18 14–18 14–18	a b c d e f g h	6–8 6–8 6–8 6–8 6–8 6–8 6–8 6–8	7–14 7–14 7–14 7–14 7–14 7–14 7–14 7–14	a b c d e f g h	6–8 6–8 6–8 6–8 6–8 6–8 6–8 6–8	7–14 7–14 7–14 7–14 7–14 7–14 7–14 7–14	a b c d e f g h i j	10≈ 10≈ 10≈ 10≈ 10≈ 10≈ 10≈ 10≈ 10≈ 10≈	7–14 7–14 7–14 7–14 7–14 7–14 7–14 7–14 7–14 7–14	a b c d e f g h i j	10≈ 10≈ 10≈ 10≈ 10≈ 10≈ 10≈ 10≈ 10≈ 10≈	7–14 7–14 7–14 7–14 7–14 7–14 7–14 7–14 7–14 7–14
	III			III			III			III			III			III			III			III			III			III		
	a c	6–8 6–8	36–48 36–48	a c	6–8 6–8	36–48 36–48	a c	6–8 6–8	36–48 36–48	a c	6–8 6–8	36–48 36–48	a b c d	6–8 6–8 6–8 6–8	16–24 16–24 16–24 16–24	a b c d	6–8 6–8 6–8 6–8	16–24 16–24 16–24 16–24	a b c d	6–8 6–8 6–8 6–8	16–24 16–24 16–24 16–24	a b c d	6–8 6–8 6–8 6–8	16–24 16–24 16–24 16–24	a b c d	10≈ 10≈ 10≈ 10≈	16–24 16–24 16–24 16–24	a b c d	10≈ 10≈ 10≈ 10≈	16–24 16–24 16–24 16–24

Note: W: Week; P: Proposal; T: S: Sets; R: Repetition; S: Session; Type of Exercise (TE); (I) Exercises combined with driving the ball: (a) zig zag between pikes; (b) passing the ball; (c) quick forwards and backwards, and (d) shooting at goal. (II) Coordination ladder exercises: (a) Single foot in each square, (b) Two feet in each square, (c) Lateral stepping, (d) Jumping jack feet, (e) In in out out, (f) Lateral Carioca, (g) Cross-overs, (h) Icey Shuffle, (i) Single foot hops ladder drill, and (j) Side Shuffle Speed Ladder]. (III) Agility multi-jump: (a) single-leg jump, (b) single-leg hop, (c) hop, and (d) jump. All the session were and progressing, varying, and experiencing difficulty by weeks. (between 70–100 jumps for session).

**Table 2:** Performance variables before (pretest) and after (posttest) the intervention period (mean ± SD).

		Young Male Soccer Players (*n* = 40)							
Control Group (*n* = 20)					Experimental Group (*n* = 20)				Differences between Groups (ANCOVA Test)
	Pretest	Posttest	SMD	RM *t*-Test (*p*)	Pretest	Posttest	SMD	RM *t*-Test (*p*)	
Yo-Yo intermittent recovery test-Level 1									
YYIR1. Distance (m)	1588 ± 242.98	**-**	**-**	**-**	1549 ± 346.52	**-**	**-**	**-**	
VO2max (ml/kg/min)	41.80 ± 0.82	**-**	**-**	**-**	41.66 ± 1.23	**-**	**-**	**-**	
Countermovement jump									
CMJ (cm)	29.95 ± 3.74	30.98 ± 4.55	1.03 ± 0.81	*p* = 0.09 | *d* = 0.25	28.83 ± 3.96	30.47 ± 4.30	1.64 ± 0.34	*p* = 0.001 | *d* = 0.50	*F*_(1,38)_ = 2251.58; *p*= 0.000; *η_p_^2^* = 0.98
Illinois agility test									
IAT (s)	16.55 ± 0.42	16.74 ± 0.68	0.19 ± 0.26	*p* = 0.12 | *d* = 0.34	16.76 ± 0.75	16.45 ± 0.72	−0.31 ± −0.03	*p* =0.002 | *d* = 0.41	*F*_(1,38)_ = 29736.3; *p* = 0.000; *η_p_^2^* = 0.99
Linear sprint									
30 m (s)	4.58 ± 0.25	4.64 ± 0.31	0.06 ± 0.06	*p* = 0.20 | *d* = 0.21	4.62 ± 0.21	4.70 ± 0.22	0.08 ± 0.01	*p* =0.21 | *d* = 0.37	*F*_(1,38)_ = 17217.1; *p* = 0.000; *η_p_^2^* = 1.00

Note: SMD, standard mean difference (posttest–pretest); RM, repeated measures.

## Data Availability

The datasets generated during and analyzed during the current study are available from the aim author or the corresponding author on reasonable request.

## References

[1] McMillan K., Helgerud J., Grant S.J., Newell J., Wilson J., Macdonald R., Hoff J. (2005). Lactate threshold responses to a season of professional British youth soccer. Br. J. Sports Med..

[2] Venturelli M., Bishop D., Pettene L. (2008). Sprint Training in Preadolescent Soccer Players. Int. J. Sports Physiol. Perform..

[3] Naughton G., Farpour-Lambert N.J., Carlson J., Bradney M., Van Praagh E. (2000). Physiological issues surrounding the performance of adolescent athletes. Sports Med..

[4] Karpowicz K., Krych K., Karpowicz M., Nowak W., Gronek P. (2018). The relationship between CA repeat polymorphism of the IGF-1 gene and the structure of motor skills in young athletes. Acta Biochim. Pol..

[5] Brughelli M., Cronin J., Levin G., Chaouachi A. (2008). Understanding change of direction ability in sport: A review of resistance training studies. Sport Med..

[6] Freitas T.T., Pereira L.A., Alcaraz P.E., Arruda A.F.S., Guerriero A., Azevedo P., Loturco I. (2019). Influence of Strength and Power Capacity on Change of Direction Speed and Deficit in Elite Team-Sport Athletes. J. Hum. Kinet..

[7] Petrigna L., Karsten B., Marcolin G., Paoli A., D’Antona G., Palma A., Bianco A. (2019). A Review of Countermovement and Squat Jump Testing Methods in the Context of Public Health Examination in Adolescence: Reliability and Feasibility of Current Testing Procedures. Front. Physiol..

[8] Warr D.M., Pablos C., Sánchez-Alarcos J.V., Torres V., Izquierdo J.M., Redondo J.C. (2020). Reliability of measurements during countermovement jump assessments: Analysis of performance across subphases. Cogent Soc. Sci..

[9] Comfort P., Stewart A., Bloom L., Clarkson B. (2014). Relationships Between Strength, Sprint, and Jump Performance in Well-Trained Youth Soccer Players. J. Strength Cond. Res..

[10] Carr C., McMahon J.J., Comfort P. (2015). Relationships between jump and sprint performance in first-class county cricketers. J. Trainology.

[11] Castillo-Rodríguez A., Fernández-García J.C., Chinchilla-Minguet J.L., Carnero E. (2012). Relationship Between Muscular Strength and Sprints with Changes of Direction. J. Strength Cond. Res..

[12] Young W.B., Dawson B., Henry G.J. (2015). Agility and Change-of-Direction Speed are Independent Skills: Implications for Training for Agility in Invasion Sports. Int. J. Sports Sci. Coach..

[13] Mota T., Afonso J., Sá M., Clemente F. (2021). An Agility Training Continuum for Team Sports: From Cones and Ladders to Small-Sided. Strength Cond. J..

[14] Jones P., Bampouras T.M., Marrin K. (2009). An investigation into the physical determinants of change of direction speed. J. Sports Med. Phys. Fit..

[15] McMahon J.J., Rej S.J.E., Comfort P. (2017). Sex Differences in Countermovement Jump Phase Characteristics. Sports.

[16] Bosco C., Komi P.V., Ito A. (1981). Prestretch potentiation of human skeletal muscle during ballistic movement. Acta Physiol. Scand..

[17] Barnes J.L., Schilling B.K., Falvo M.J., Weiss L.W., Creasy A.K., Fry A.C. (2007). Relationship of Jumping and Agility Performance in Female Volleyball Athletes. J. Strength Cond. Res..

[18] Vescovi J.D., McGuigan M. (2008). Relationships between sprinting, agility, and jump ability in female athletes. J. Sports Sci..

[19] Meylan C., McMaster T., Cronin J., Mohammad N.I., Rogers C., Deklerk M. (2009). Single-Leg Lateral, Horizontal, and Vertical Jump Assessment: Reliability, Interrelationships, and Ability to Predict Sprint and Change-of-Direction Performance. J. Strength Cond. Res..

[20] Paul D.J., Gabbett T.J., Nassis G.P. (2015). Agility in Team Sports: Testing, Training and Factors Affecting Performance. Sports Med..

[21] Darby S.A., Frysztak R.J. (2014). Neuroanatomy of the Spinal Cord. Clinical Anatomy of the Spine, Spinal Cord, and ANS.

[22] Marković G., Sekulic D., Marković M. (2007). Is agility related to strength qualities?—Analysis in latent space. Coll. Antropol..

[23] Bianco A., Jemni M., Thomas E., Patti A., Paoli A., Roque J.R., Palma A., Mammina C., Tabacchi G. (2015). A systematic review to determine reliability and usefulness of the field-based test batteries for the assessment of physical fitness in adolescents—The ASSO Project. Int. J. Occup. Med. Environ. Health.

[24] Zouhal H., Abderrahman A.B., Dupont G., Truptin P., Le Bris R., Le Postec E., Sghaeir Z., Brughelli M., Granacher U., Bideau B. (2019). Effects of Neuromuscular Training on Agility Performance in Elite Soccer Players. Front. Physiol..

[25] Morat M., Morat T., Zijlstra W., Donath L. (2021). Effects of multimodal agility-like exercise training compared to inactive controls and alternative training on physical performance in older adults: A systematic review and meta-analysis. Eur. Rev. Aging Phys. Act..

[26] De Blas X., Padullés J.M., Del Amo J.L.L., Guerra-Balic M. (2012). Creation and Validation of Chronojump-Boscosystem: A Free Tool to Measure Vertical Jumps. Rev. Int. Cienc. Deporte..

[27] Samozino P., Rabita G., Dorel S., Slawinski J., Peyrot N., De Villarreal E.S., Morin J.-B. (2015). A simple method for measuring power, force, velocity properties, and mechanical effectiveness in sprint running. Scand. J. Med. Sci. Sports.

[28] Romero-Franco N., Jiménez-Reyes P., Castaño-Zambudio A., Capelo-Ramírez F., Rodríguez-Juan J.J., González-Hernández J., Toscano-Bendala F.J., Cuadrado-Peñafiel V., Balsalobre-Fernández C. (2016). Sprint performance and mechanical outputs computed with an iPhone app: Comparison with existing reference methods. Eur. J. Sport Sci..

[29] Bond C.W., Willaert E.M., Rudningen K.E., Noonan B.C. (2017). Reliability of Three Timing Systems Used to Time Short on Ice-Skating Sprints in Ice Hockey Players. J. Strength Cond. Res..

[30] Amiri-Khorasani M., Abu Osman N.A., Yusof A. (2011). Acute effect of static and dynamic stretching on hip dynamic range of motion during instep kicking in professional soccer players. J. Strength Cond. Res..

[31] Daneshjoo A., Mokhtar A.H., Rahnama N., Yusof A. (2012). The effects of comprehensive warm-up programs on proprioception, static and dynamic balance on male soccer players. PLoS ONE.

[32] Kilding A.E., Tunstall H., Kuzmic D. (2008). Suitability of FIFA’s “The 11” Training Programme for Young Football Players—Impact on Physical Performance. J. Sports Sci. Med..

[33] Sporiš G., Milanovićl L., Jukićl I., Omrčen D., Molinuevo J.S. (2010). The effect of agility training on athletic power performance. Kinesiology.

[34] Jovanovic M., Sporis G., Omrčen D., Fiorentini F. (2011). Effects of Speed, Agility, Quickness Training Method on Power Performance in Elite Soccer Players. J. Strength Cond. Res..

[35] Padrón-Cabo A., Rey E., Kalén A., Costa P.B. (2020). Effects of Training with an Agility Ladder on Sprint, Agility, and Dribbling Performance in Youth Soccer Players. J. Hum. Kinet..

[36] Asadi A., Arazi H., Ramirez-Campillo R., Moran J., Izquierdo M. (2017). Influence of Maturation Stage on Agility Performance Gains After Plyometric Training: A Systematic Review and Meta-analysis. J. Strength Cond. Res..

[37] Kovacikova Z., Zemková E. (2020). The Effect of Agility Training Performed in the Form of Competitive Exercising on Agility Performance. Res. Q. Exerc. Sport.

[38] Andrzejewski M., Chmura J., Pluta B., Konarski J.M. (2015). Sprinting Activities and Distance Covered by Top Level Europa League Soccer Players. Int. J. Sports Sci. Coach..

[39] Aloui G., Hermassi S., Hayes L., Hayes N.S., Bouhafs E., Chelly M., Schwesig R. (2021). Effects of Plyometric and Short Sprint with Change-of-Direction Training in Male U17 Soccer Players. Appl. Sci..

[40] Sander A., Keiner M., Wirth K., Schmidtbleicher D. (2013). Influence of a 2-year strength training programme on power performance in elite youth soccer players. Eur. J. Sport Sci..

[41] Uzelac-Sciran T., Sarabon N., Mikulic P. (2020). Effects of 8-Week Jump Training Program on Sprint and Jump Performance and Leg Strength in Pre- and Post-Peak Height Velocity Aged Boys. J. Sports Sci. Med..

[42] Makhlouf I., Chaouachi A., Chaouachi M., Ben Othman A., Granacher U., Behm D.G. (2018). Combination of Agility and Plyometric Training Provides Similar Training Benefits as Combined Balance and Plyometric Training in Young Soccer Players. Front. Physiol..

